# Assessing the Effects of Data Selection and Representation on the Development of Reliable *E. coli* Sigma 70 Promoter Region Predictors

**DOI:** 10.1371/journal.pone.0119721

**Published:** 2015-03-24

**Authors:** Mostafa M. Abbas, Mostafa M. Mohie-Eldin, Yasser EL-Manzalawy

**Affiliations:** 1 KINDI Center for Computing Research, College of Engineering, Qatar University, Doha, Qatar; 2 Department of Mathematics, Faculty of Science, Al-Azhar University, Cairo, Egypt; 3 Systems and Computer Engineering, Al-Azhar University, Cairo, Egypt; 4 College of Information Sciences, Penn State University, University Park, United States of America; International Centre for Genetic Engineering and Biotechnology (ICGEB), INDIA

## Abstract

As the number of sequenced bacterial genomes increases, the need for rapid and reliable tools for the annotation of functional elements (e.g., transcriptional regulatory elements) becomes more desirable. Promoters are the key regulatory elements, which recruit the transcriptional machinery through binding to a variety of regulatory proteins (known as sigma factors). The identification of the promoter regions is very challenging because these regions do not adhere to specific sequence patterns or motifs and are difficult to determine experimentally. Machine learning represents a promising and cost-effective approach for computational identification of prokaryotic promoter regions. However, the quality of the predictors depends on several factors including: i) training data; ii) data representation; iii) classification algorithms; iv) evaluation procedures. In this work, we create several variants of E. coli promoter data sets and utilize them to experimentally examine the effect of these factors on the predictive performance of E. coli *σ* ^70^ promoter models. Our results suggest that under some combinations of the first three criteria, a prediction model might perform very well on cross-validation experiments while its performance on independent test data is drastically very poor. This emphasizes the importance of evaluating promoter region predictors using independent test data, which corrects for the over-optimistic performance that might be estimated using the cross-validation procedure. Our analysis of the tested models shows that good prediction models often perform well despite how the non-promoter data was obtained. On the other hand, poor prediction models seems to be more sensitive to the choice of non-promoter sequences. Interestingly, the best performing sequence-based classifiers outperform the best performing structure-based classifiers on both cross-validation and independent test performance evaluation experiments. Finally, we propose a meta-predictor method combining two top performing sequence-based and structure-based classifiers and compare its performance with some of the state-of-the-art E. coli *σ* ^70^ promoter prediction methods.

## Introduction

Transcription initiation is the first and key step leading to gene expression [1]. The process starts with the binding of RNA polymerase (RNAP) to a specific segment in DNA (called promoter region) located upstream of the transcription start site (TSS). Understanding how RNAP locates and recognize promoter regions remains an active research area in molecular biology and a challenging task in bioinformatics. In bacteria, transcription initiation requires an additional subunit called *σ* factor, which associates with the core RNA polymerase to form a holoenzyme [2, 3]. Different *σ* factors interact with distinct consensus promoter sequences. Each *σ* factor is labeled according to its molecular weight (e.g., *σ* ^24^, *σ*,^28^ *σ* ^32^, *σ* ^38^, *σ* ^54^, and *σ* ^70^). The accurate identification of *σ*-specific promoter regions is tiresome and technically exacting [4–6]. Therefore, computational methods for reliably identifying promoter sequences are highly desirable.

Many computational methods for predicting promoter regions in prokaryotes have been proposed in literature (e.g., [7–24]). Among these methods, several machine learning algorithms have been used in developing prokaryotic promoter region prediction methods. For example, support vector machine (SVM) [7, 8, 12, 25, 26], artificial neural networks (ANNs) [16, 17, 20, 27–29], partial least square (PLS) [18], and quadratic discriminant analysis (QDS) [14]. Some methods are based on probabilistic approaches (e.g., hidden Markov models (HMMs) [30] and a combination of HMMs and ANNs [31]). In such prediction methods, the promoter identification problem is viewed as a binary classification problem such that the given test sequence is predicted to be a promoter or a non-promoter [27].

In general, the quality of these prediction methods depends on several factors including: i) Training data: Does the data include redundant sequences? Is negative data experimentally validated? If not, how is negative data generated?; ii) Data representation: The vast majority of machine learning algorithms do not accept DNA sequence as input. Hence, some technique has to be applied to map each sequence into a vector of (often numeric) features such that the machine learning algorithm can efficiently discriminate between positively labeled and negatively labeled sequences; iii) Classification algorithms: Typically, the developer has to apply several machine learning algorithms to the target data and use the one with the best performance as the final predictor; iv) Evaluation procedures: There exist two widely-used evaluation procedures, cross-validation and blind test evaluations. In k-fold cross-validation experiments, the data is randomly partitioned into *k* subsets of equal size. Then, *k* − 1 folds are used to train the classifier and the hold away fold is used for testing. This step is repeated *k* times such that in each time a different fold is hold for testing the classifier. Leave-one-out evaluation procedure is a cross-validation procedure where *k* is set to the number of instances in the data. In blind test set experiments, an independent test set is prepared and used for evaluating the trained classifiers.

For each factor, several design choices have been made by the developers of prokaryotic promoter regions prediction methods. Following are some examples: i) Training data: Due to lack of sufficient data, the vast majority of methods did not remove redundant data (i.e., promoters or non-promoter sequences that share high similarity scores). No experimentally validated non-promoter sequence data source exists. Therefore, developers have to generate their non-promoter sequences. Several strategies for generating non-promoter sequences have been used including: randomly generated sequences [16, 17, 28]; sequences extracted from intergenic or coding regions [7, 11, 12, 14, 15, 18, 25, 28]; ii) Feature extraction: Several sequence and structure-based feature representations have been used for developing prokaryotic promoter region prediction methods. Examples of sequence based features include: k-mer representation [7, 12, 28, 32], variable-window Z-curve [18], and nucleotide identity (NID) [17]. Examples of structure based features include: stress induced duplex destabilization (SIDD), DNA curvature and stacking energy explored in [13], roll, tilt, twist and average free energy used in [14], and DNA stability proposed in [23]; iii) Classification algorithms: support vector machines (SVMs) and artificial neural networks (ANNs) are widely used for this classification task; iv) Evaluation procedures: the vast majority of prediction methods [7, 8, 10, 12, 13, 15–17, 19, 26, 33] have been evaluated using cross-validation experiments. However, few methods (e.g., [11, 15]) employed blind test sets in addition to cross-validation procedure to assess the performance of their predictors.

Against this background, we conduct extensive experiments to analyze the influence of these factors on prokaryotic promoter region predictors. One of our major aims is to guide the developers of future prokaryotic promoter region predictors to make appropriate design decisions (e.g., how to generate non-promoter sequences? how to get an accurate estimate of the performance of your classifier? how to avoid misleading conclusions?). Our results suggests that good representative non-promoter sequences should be extracted using multiple strategies (e.g., a combination of randomly generated sequences and sequences extracted from coding and non-coding regions). Our results also demonstrate that cross-validation estimates might be misleading (especially, when the non-promoter sequences are randomly generated or extracted from coding regions). We show that a more accurate estimate of performance could be obtained using high-quality independent test set or by averaging over multiple versions of the cross-validation data. Finally, we propose a meta-predictor combining two sequence-based and structure-based predictors for predicting E. coli *σ* ^70^ promoter regions and compare it with some state-of-the-art prediction methods.

## Materials and Methods

### Data sets

We used experimentally validated promoters from E. coli, a well studied prokaryotic model organism. RegulonDB [34, 35] is a rich resource for curated information on transcriptional regulation in E. coli K-12. The latest version, RegulonDB 8, has been enriched with a large number of promoters and TSS mapped using high-throughput technology. In our experiments, we used promoters extracted from RegulonDB 7 for constructing our cross-validation data sets and promoters extracted from RegulonDB 8 but not in RegulonDB 7 to construct our independent test sets. We limited our experiments to *σ* ^70^ promoters due to the lack of sufficient training for other *σ* factors dependent data sets after removing redundant sequences. Both cross-validation and test sets are included in the Supporting Information section (S1 Dataset).

#### Generation of negative data sets

Like many bioinformatics classification tasks, predicting prokaryotic promoter region is challenged by the lack of experimentally validated negative (i.e., non-promoter) data. To study how different approaches for generating non-promoter sequences might affect the performance of the classifier, we explored three approaches that randomly select non-promoter regions from: i) Randomly generated DNA sequences. DNA segments of length 81 were randomly selected from a DNA sequence of length 1000,000 that was randomly generated with frequencies 0.28, 0.22, 0.22, and 0.28 for T, G, C, and A (respectively); ii) Coding regions in E. coli K12 genome downloaded from NCBI GenBank [36]; iii) Intergenic regions in E. coli K12 genome downloaded from Ecogene database [37] and categorized into convergent, divergent, Codirectional+, and Codirectional-.

#### Cross-validation data sets

We extracted 741 *σ* ^70^ promoters from RegulonDB 7. After removing sequences that share more than 45% similarity, our final set of positive data consists of 579 promoter sequences. Seven versions of the cross-validation data set has been constructed by combining the positive set with seven non-redundant, by removing sequences that share more than 45% similarity, sets of 579 sequences (none of them share more than 45% sequence similarity with any positive sequence). To the best of our knowledge, this is the first E. coli promoter region data set that establishes some criteria for reducing sequence similarity. Although a more stringent similarity cutoff might be preferred, this choice was not applicable as the number of the promoter sequences drops to 100 sequences at 35% similarity cutoff. The seven versions of the cross-validation data share the same positive set but each data set version has different negative set (see Table 1).

**Table 1:** Summary of cross-validation data sets.

**Data set**	**Source of negative data**
CV_Random	Randomly extracted from a single long sequence that is generated with frequencies 0.28, 0.22, 0.22, and 0.28 for T, G, C and A (respectively), according to Silva et al., [17]
CV_Coding	Randomly extracted from coding regions extracted form E.coli K-12 genome downloaded from NCBI GenBank [36]
CV_Convergent	Randomly extracted from convergent intergenic regions downloaded from EcoGene 3.0 database [37]
CV_Divergent	Randomly extracted from divergent intergenic regions downloaded from EcoGene 3.0 database [37]
CV_CoPos	Randomly extracted from codirectional positive spacer regions downloaded from EcoGene 3.0 database [37]
CV_CoNeg	Randomly extracted from codirectional negative spacer regions downloaded from EcoGene 3.0 database [37]
CV_Mixed	Six equal subsets of negative sequences extracted from negative sequences in CV_Random, CV_Coding, CV_Convergent, CV_Divergent, CV_CoPos, and CV_CoNeg

#### Independent test sets

We downloaded 1790 *σ* ^70^ promoters from RegulonDB 8. Then, we discarded from this list of sequences any sequence that share more than 45% sequence similarity with at least one promoter sequence in the cross-validation data. Our final independent test sets consist of seven versions: TS_Random; TS_Coding; TS_Convergent; TS_Divergent; TS_CoPos; TS_CoNeg, and TS_Mixed. All versions share the same 792 promoter sequences but each one has its own negative set of 792 non-promoters generated using the same procedure used with the cross-validation data. None of the negative data sequences share more than 45% sequence similarity with any corresponding negative cross-validation data set sequence.

### Features extraction

In our data sets, each promoter sequence is 81 bp long region [TSS-60…TSS+20], with the mapped TSS at position 0. Non-promoter sequences are also 81 pb long regions with no validated TSS at position 0. The vast majority of machine learning algorithms can not be applied directly to such input. Instead, a per-processing step (called features extraction) has to be performed in order to map each DNA sequence into a feature vector. For instance, 1-mer features representation maps each DNA sequences into four numeric features, that are typically the frequency of the four types of nucleotides in the target DNA sequence.

In our experiments, we evaluated several features extraction methods, which have been widely used for promoter classification tasks (e.g., [7, 12, 14, 28, 32]) and for DNA classification tasks (e.g., [38–40]). The features extraction methods that we evaluated could be categorized into two main categories: i) sequence-based features; ii) structure-based features.

For sequence-based features, we used k-mer features [7, 12, 28, 32] with k = 1, 2, …, 5. k-mer features representation maps each DNA sequence into 4^*k*^ numeric features representing the normalized counts of each k-mer substring in the sequence. A major limitation of k-mer features is that some sequence order information is lost. We evaluated two sequence-based features that preserves the sequence order information: i) nucleotide identity (NID) features; ii) dinucleotides identity (DNID) features. In nucleotide identity features, each 81 nucleotides long DNA sequence is represented with 81 features. Each feature is a nominal attribute which can take any value from the set {A, C, G, T}. In dinucleotides identity features, each 81 nucleotides long DNA sequence is represented with 80 features. Each feature is a nominal attribute which can take any value from the set {AA, AC, AG, AT, CA, CC, CG, CT, GA, GC, GG, GT, TA, TC, TG, TT}. It should be noted that some classifiers (e.g., Naive Bayes and Random Forests) works directly with nominal (i.e., categorical) attribute values, while others (e.g., Support Vector Machines and Neural Networks) do not support this type of attributes and requires the conversion into numeric features (i.e., using orthogonal codification to convert each nominal value into a binary string).

For structure-based features, we evaluated several DNA structure-based features derived from twelve dinucleotides scales including: A-philicity (M1) [41]; Ohler B-DNA twist (M2) [42]; Olson B-DNA twist (M3) [43]; DNA bending stiffness (M4) [44]; DNA denaturation temperature (M5) [45]; Z-DNA free energy (M6) [46]; duplex disruption free energy (M7) [47]; duplex stability free energy (M8) [48]; protein-induced deformability (M9) [43]; propeller twist (M10) [49]; protein-induced DNA twist (M11) [43]; and base stacking energy (M12) [50]. Using these scales, each DNA sequence in our data is represented with 80 numeric features using the procedure described in [51] and a smoothing window of size equals three.

All sequence and structure-based features are implemented as part of the Genome Annotation Toolkit (Gennotate) [52]. Gennotate is an extension of WEKA [53], a widely used machine learning workbench supporting many standard machine learning algorithms. Most of these algorithms could not be applied directly to DNA sequence data sets. Developers have to pre-process their data for feature extraction and then apply WEKA implemented algorithms to the data set in its numerical representation. Gennotate integrates the DNA feature extraction step into WEKA and allows on-the-fly mapping of DNA sequences into feature vectors. This simplifies and expedites the process of building machine learning based models for different genome annotation tasks and facilitates sharing offline versions of developed models and the development of consensus and hybrid models on top of existing ones [52].

### Classification algorithms

We evaluated three machine learning algorithms that are widely used in bioinformatics sequence classification tasks: i) Naive Bayes (NB) [54]; ii) Random Forest (RF) [55]; iii) Support Vector Machines (SVM) [56]. Each of these algorithms has some strengths and weaknesses. For example, NB is superior in terms of training speed, training simplicity (i.e., no parameter tuning is needed), and scalability. On the other hand, NB algorithm relies on a strong assumption regarding attribute independence. We discuss the strengths and limitations of each algorithm in predicting E. coli *σ* ^70^ promoters in the Results and Discussion section. In the following paragraphs, we summarize the three algorithms.

The NB classifier [54] is a direct and straightforward application of Bayes Theorem. The main assumption of NB classifier is the conditional independence of all attributes given the class label. In spite of the unrealistic assumption of independence, the performance of NB classifier is competitive with sophisticated classifiers for many real-world classification tasks. The NB classifier takes the random variable *X* = (*x*_1_, *x*_2_, *x*_3_, ….., *x*_*n*_), promoter sequence features, as input and produce the binary class *C* ∈ {1, 0} as output, where ‘1’ denotes a promoter and ‘0’ denotes a non-promoter. For a query instance, *X*, NB classifier returns ‘1’ (promoter) if: $\frac{P(C=1|X=x_{1},x_{2},x_{3},.....,x_{n})}{P(C=0|X=x_{1},x_{2},x_{3},.....,x_{n})}=\frac{P(C=1)\prod_{i=1}^{n}P(x_{i}=x|C=1)}{P(C=0)\prod_{i=1}^{n}P(x_{i}=x|C=0)}\geq 1$ and returns the class label ‘0’ (non-promoter) otherwise.

The RF classifier [55] is a combination of random decision tree base classifiers. It integrates *bagging* [57] with the random selection of subset feature for training decision trees as following: i) Generating bootstrap samples with *n* training instances (i.e., randomly selecting with replacement *n* instances from the training data); ii) Randomly selecting *m* variables from the set of *M* input features, *m* ≪ *M*, and using the sampled training data for generating individual base decision trees. The *k* tree classifiers will be constructed by repeating this two-step procedure. The RF classifier reports the average prediction of all decision tree classifiers for given query instance. In our experiments, we used RF classifier with *k* = 100.

The SVM classifier [56] is based on the concept of decision planes that define decision boundaries. The SVM classifier maps the input features into feature vectors in a high-dimensional feature space. In the training stage, the data is separated into positive and negative in the feature space by finding a hyperplane that maximizes the margin of separation. In case of non-linearly separable training data in the input space, SVM classifier uses a kernel function *K* to map non-linearly separable data in the input space into a typically high-dimensional feature space where the data are assumed to be linearly separable without explicitly mapping each training example from the input space into the feature space. The selection of the kernel function is a critical factor in training SVM classifiers. Therefore, the performance of SVM classifier depends on selecting a suitable kernel and tuning the kernel parameters (if any). In our experiments, we applied two widely-used kernel functions: i) Linear kernel; ii) Radial bias kernel (RBF).

The input of the above classifiers is a variety of sequence or structural features of the promoter and non-promoter sequences. On the other hand, we tried an additional classifier (based on HMM algorithm [58]) that takes the DNA sequence itself as input. The HMM classifier is a stochastic generative model classifier based on Markov chain. The HMM classifier assumes that labels are hidden and its goal is to predict these hidden labels given the input sequence. The HMM is composed of two stochastic processes. The first process is characterized by hidden states (with three types: match, delete, and insert states) and probabilities of transition such that each state depends only on the previous state (i.e., Markov property is established). The second process produces emissions observable at each moment, based on a state-dependent probability distribution. The parameters of an HMM will be iteratively modified during the training phase. We used a java implementation of HMM algorithm that is provided in Gennotate tool [52].

### Performance evaluation

The predictive performance of promoter region prediction classifiers was assessed using Accuracy (ACC), Sensitivity (*S*_*n*_), Specificity (*S*_*p*_), and Mathew Correlation Coefficient (*MCC*) measures defined as follows [59, 60]:

$$\begin{array}{c} ACC=\frac{TP+TN}{TP+FP+TN+FN} \end{array}$$ (1)

$$\begin{array}{c} S_{n}=\frac{TP}{TP+FN} \end{array}$$ (2)

$$\begin{array}{c} S_{p}=\frac{TN}{TN+FP} \end{array}$$ (3)

$$\begin{array}{c} MCC=\frac{TP\times TN-FP\times FN}{\sqrt{\left(TN+FN)(TN+FP)(TP+FN)(TP+FP\right)}} \end{array}$$ (4)

where TP, FP, TN, and FN are the numbers of true positive(promoter sequence classified as promoter), false positive(non-promoter sequence classified as promoter), true negative(non-promoter sequence classified as non-promoter), and false negative(promoter sequence classified as non-promoter), respectively.

All these metrics are determined using a specific threshold value, which could be viewed as a trade off between *S*_*n*_ and *S*_*p*_. The Receiver Operating Characteristic (ROC) curve [61] provides a wide comprehensive picture of the performance of the predictor where it describes the performance of the classifier over all possible thresholds. The ROC curve is a two-dimensional graph in which the true positive rate is plotted on the *Y* axis and the false positive rate is plotted on the *X* axis. Each point on the ROC curve represents the behavior of the classifier at a specific choice threshold value, and hence a particular choice of tradeoff between true positive rate and false positive rate. The area under ROC curve (AUC) is equivalent to the probability that a randomly chosen positive example will be ranked higher than a randomly chosen negative example. Swets [62] suggested evaluation grades for the classifiers based on AUC scores (see Table 2) Here, we limit our discussion to the AUC metric and report the performance of classifiers using threshold-dependent metrics in the Supporting Information section (S1 Text).

**Table 2:** Grading scale for classifiers based on their AUC scores.

AUC score	Grade
0.90–1.00	Excellent
0.80–0.89	Good
0.70–0.79	Fair
0.50–0.69	Poor

### Statistical analysis

For comparing several classifiers on multiple data sets, we used the non-parametric statistical test proposed by Demšar [63]. First, classifiers are ranked based on their observed performance (e.g., AUC) for each data set separately (i.e., for each data set, the best classifier is assigned a rank of 1, the second best classifier is ranked 2, and so on). Second, the Friedman test is applied to determine whether the measured average ranks are significantly different from the mean rank under the null hypothesis. Third, if the null hypothesis can be rejected at a significance level of 0.05, the Nemenyi test is used to determine whether significant differences exist between any given pair of classifiers.

## Results and Discussion

### Misleading cross-validation performance estimates

Tables S1-S5 in S1 Text report the average AUC of several sequence-based classifiers obtained using the average of 10 runs of 10-fold cross-validation experiments. Classifiers with excellent performance (*AUC* ≥ 0.90) are observed more oftenly when classifiers are evaluated using CV_Random and CV_Coding data sets. However, such classifiers with excellent (or good) cross-validation performance estimates might perform poorly in real world scenarios. For example, the classifier NB_3-mer_Coding has AUC score equals 0.91. However, when this classifier is evaluated using TS_Coding, TS_Convergent, and TS_Mixed test sets its AUC scores are 0.90, 0.59, and 0.68 (respectively). Another example is the classifier NB_4-mer_Random, which has AUC score equals 0.87 while its performance on TS_Coding, TS_Convergent, and TS_Mixed test sets is 0.91, 0.59, and 0.68 (respectively).

For structure-based classifiers (Tables S6-S9 in S1 Text), the performance obtained using CV_Coding data set is significantly higher than the performance of the classifiers evaluated on other versions of the cross-validation data (including CV_Random). These results suggest that, for cross-validation experiments, there exists some combination of classifier design choices (e.g., randomly generated negative data combined with k-mer features) that could produce a classifier with excellent performance estimates. However, this classifier will perform poorly on independent test sets. This finding underscores the necessity of employing independent test sets for evaluating methods for predicting prokaryotic promoters.

### A hypothesis for identifying good predictors

A careful examination of results reported in Tables S1-S9 (S1 Text) suggests the following hypothesis for identifying good predictors (i.e., predictors which perform well on cross-validation and independent test experiments): A good predictor is a predictor that performs well on cross-validation data regardless how negative data is generated. In our experiments, we used the average AUC over the seven cross-validation data sets to indicate the overall classifier performance. We also used the standard deviation (STD) to indicate how sensitive the classifier to different choices of negative data. Using this hypothesis, we chose the following representative set of good predictors: NB_DNID, RF100_M7, and HMM which have *AUC* ± *STD* equal 0.85 ± 0.04, 0.80 ± 0.04, and 0.82 ± 0.05 (respectively). We also chose the following representative set of bad performing classifiers: NB_4-mer and NB_M1 with *AUC* ± *STD* equal 0.73 ± 0.12 and 0.70 ± 0.09 (respectively). Table 3 shows the performance of these five representative classifiers trained using CV_Mixed data and tested on the seven independent set versions. Interestingly, all classifiers could discriminate between promoter and coding sequences. Another interesting observation is that the AUC using TS_Mixed is within ±0.03 of the AUC obtained using CV_Mixed cross-validation data set. This suggest that the average cross-validation performance estimate obtained using different versions of the cross-validation data, created using different techniques for generating non-promoter sequences, is a good estimate of performance estimates obtained on independent test sets.

**Table 3:** AUC scores for selected classifiers (trained using CV_Mixed data) and tested on different versions of independent test set (e.g., TS_Random and TS_Coding).

**Data set**	**NB_DNID**	**RF100_M7**	**HMM**	**NB_4-mer**	**NB_M1**	**meta-predictor**
TS_Random	0.83(1.5)	0.77(5.0)	0.80(3.5)	0.76(6.0)	0.80(3.5)	0.83(1.5)
TS_Coding	0.89(2.5)	0.87(5.0)	0.89(2.5)	0.88(4.0)	0.86(6.0)	0.91(1.0)
TS_Convergent	0.80(2.5)	0.80(2.5)	0.78(4.0)	0.64(6.0)	0.66(5.0)	0.82(1.0)
TS_Divergent	0.80(2.0)	0.79(3.0)	0.78(4.0)	0.61(6.0)	0.65(5.0)	0.82(1.0)
TS_CoPos	0.79(2.0)	0.78(3.0)	0.76(4.0)	0.58(6.0)	0.66(5.0)	0.81(1.0)
TS_CoNeg	0.82(2.0)	0.80(3.5)	0.80(3.5)	0.68(5.5)	0.68(5.5)	0.84(1.0)
TS_Mixed	0.83(2.0)	0.82(3.0)	0.81(4.0)	0.70(6.0)	0.71(5.0)	0.85(1.0)
Average	0.82(2.0)	0.80(3.4)	0.80(3.5)	0.69(5.5)	0.72(4.9)	0.84(1.1)
STD	0.03	0.03	0.04	0.10	0.08	0.03

See Methods Section for more information about these test sets. For each data set, the rank of each classifier is shown in parentheses.

### Sequence-based versus structure-based predictors

Tables S1-S4 (S1 Text) show that sequence-based classifiers evaluated using NID and DNID features representation outperform classifiers evaluated using k-mer features representation (in terms of higher AUC scores and lower standard deviations). The top two classifiers are SVMRBF_DNID and NB_DNID with *AUC* ± *STD* equal 0.86 ± 0.05, 0.85 ± 0.04 (respectively).

Tables S6-S9 (S1 Text) show that the top performing structure-based classifiers (with *AUC* = ∼ 0.80) are obtained using Random Forest algorithm and DNA bending stiffness (M4), duplex disruption free energy (M7), duplex stability free energy (M8), or base stacking energy (M12) features representation. Interestingly, the vast majority of structure-based classifiers seem to be less sensitive to the design choice of the non-promoter sequences.

Although the cross-validation experiments suggest a superior performance of sequence-based classifiers over structure-based ones, results on independent test sets (see Table 3) narrow the gap in performance between top performing sequence-based and structure-based classifiers. A meta-predictor combining NB_DNID and RF100_M7, using average of predicted probabilities, results in 0.02 improvement in AUC over NB_DNID. Further improvements in performance could be achieved by: i) including more divergent base classifiers (e.g., HMM or classifiers using the same feature representation but different classification algorithms); ii) using more sophisticated approaches for combining base classifiers (e.g., using second stage meta-classifier). An alternative approach for integrating DNA sequence and structure-based features is to concatenate them and train a single classifier. For example, a novel DNA feature representation, pseudo dinucleotide composition, combines dinucleotide composition with six local DNA structure properties into a single feature vector has been proposed [64]. Pseudo k-tuple nucleotide composition combines k-tuple (k-mer) features with DNA structure features [65].

**Fig 1:**
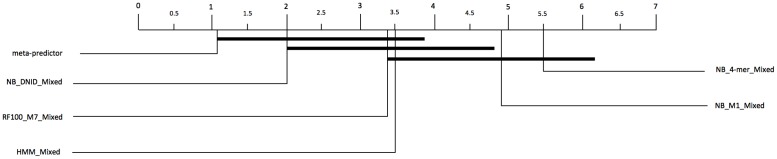
Pair-wise comparison of selected classifiers with Nemenyi test applied to results on independent test data sets. Groups of classifiers that are not significantly different (at p-value = 0.05) are connected.

For identifying the statistically significant differences in the performance of selected classifiers, we applied Demšar’s three-step procedure to the results obtained on the independent test data sets. Table 3 shows the AUC scores associated with the rank for each classifier on each data set and the average of them. At a significance of 0.05, the application of Friedman test suggests the existence of statistically significant differences between the selected methods. Hence, there is at least two classifiers such that the difference between their average ranks is statistically significant at 0.05 level of significance. The significantly different pair-wise comparisons, obtained using Nemenyi test, are summarized in Fig. 1.

### Analysis of discriminative features

**Fig 2:**
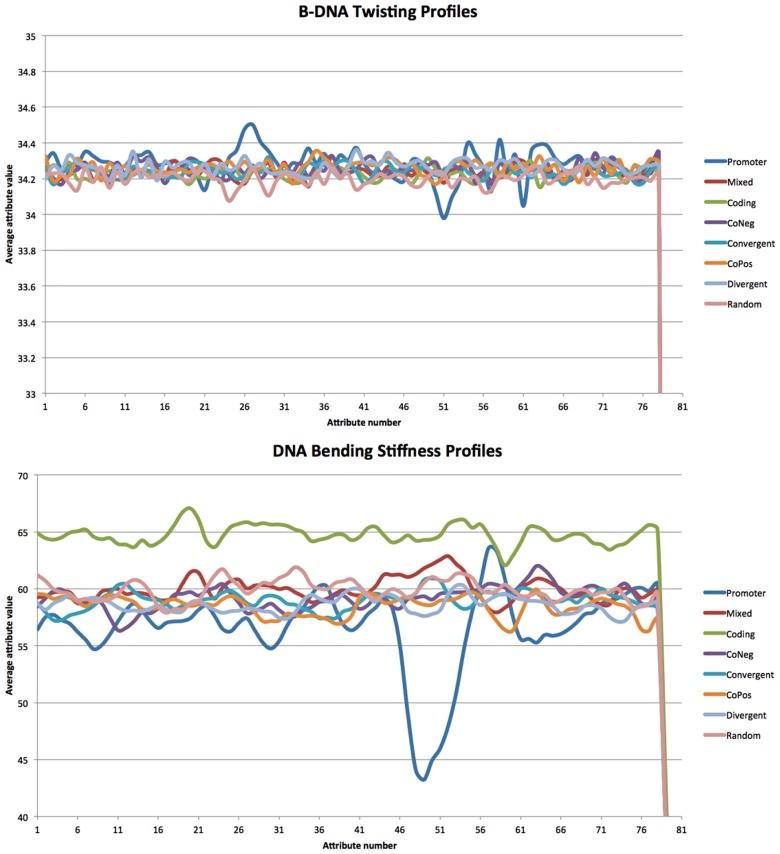
B-DNA twisting profiles (top) and DNA bending stiffness profiles (bottom) generated from cross-validation data.

Table 4 summarizes the performance of NB, RF100, SVMLnr, and SVMRBF classification methods using 12 different structure-based representations of the cross-validation data set. We observed that Olson B-DNA twist (M3) representation leads to consistent (*STD* = 0.00) average poor performance (0.5 ≤ *AUC* ≤ 0.69) while DNA bending stiffness (M4) representations leads to consistent (*STD* = 0.01) average fair performance (0.7 ≤ *AUC* ≤ 0.79). Therefore, for each of the four classification methods, M3 representation leads to a classifier with poor performance while M4 representation leads to a classifier with fair performance. To understand the differences between these two representations, we used the cross-validation data to plot the profiles for M3 and M4 (see Fig. 2). Briefly, cross-validation data (using M3 and M4 representation, respectively) were grouped into 8 groups: Promoter, Mixed, Coding, CoNeg, Convergent, CoPos, Divergent, and Random. For each group, we got a profile by averaging the values in each attribute feature. Fig. 2 shows the profiles for promoter and non-promoter sequences using M3 (top) and M4 (bottom) feature representations. Interestingly, using M4 feature representation, the profile of negative coding data can be easily discriminated from other profiles. Also, the profile of promoter data has distinguishable peak signal in the region 45–55 which allows for discriminating promoter profile from other non-promoter profiles. To validate this observation, we applied WEKA’s InfoGainAttributeEval feature selection method to rank the attributes in CV_Mixed data set using 10-fold cross-validation experiment. The top 10 ranked attributes are attributes corresponding to positions: 49, 48, 50, 51, 52, 47, 53, 54, 29, and 46. Interestingly, 9 out of top 10 ranked attributes lie in the region 45–55. On the other hand, the signal for discriminating the promoter profile from other profiles using M3 feature representation is not as strong as the one using the M4 feature representation.

**Table 4:** Summary of the performance of NB, RF100, SVMLnr, and SVMBRF classifiers on cross-validation data using twelve different structure-based feature representations.

**Features**	**NB**	**RF100**	**SVM(Lnr)**	**SVM(RBF)**	**Average**	**STD**
M1	0.70(9.0)	0.72(9.0)	0.67(11.0)	0.68(10.0)	0.69(9.8)	0.02
M2	0.65(12.0)	0.70(10.0)	0.61(12.0)	0.61(12.0)	0.64(11.5)	0.04
M3	0.68(10.5)	0.68(11.5)	0.69(9.5)	0.68(10.0)	0.68(10.4)	0.00
M4	0.78(1.5)	0.80(2.5)	0.78(1.5)	0.78(2.0)	0.79(1.9)	0.01
M5	0.76(5.5)	0.78(7.0)	0.76(5.0)	0.76(6.0)	0.77(5.9)	0.01
M6	0.78(1.5)	0.79(5.5)	0.77(3.0)	0.78(2.0)	0.78(3.0)	0.01
M7	0.74(7.5)	0.80(2.5)	0.76(5.0)	0.77(4.5)	0.77(4.9)	0.03
M8	0.77(3.5)	0.80(2.5)	0.78(1.5)	0.78(2.0)	0.78(2.4)	0.01
M9	0.74(7.5)	0.79(5.5)	0.71(7.5)	0.72(7.5)	0.74(7.0)	0.04
M10	0.76(5.5)	0.77(8.0)	0.71(7.5)	0.72(7.5)	0.74(7.1)	0.03
M11	0.68(10.5)	0.68(11.5)	0.69(9.5)	0.68(10.0)	0.68(10.4)	0.00
M12	0.77(3.5)	0.80(2.5)	0.76(5.0)	0.77(4.5)	0.78(3.9)	0.02

A-philicity (M1) [41]; Ohler B-DNA twist (M2) [42]; Olson B-DNA twist (M3) [43]; DNA bending stiffness (M4) [44]; DNA denaturation temperature (M5) [45]; Z-DNA free energy (M6) [46]; duplex disruption free energy (M7) [47]; duplex stability free energy (M8) [48]; protein-induced deformability (M9) [43]; propeller twist (M10) [49]; protein-induced DNA twist (M11) [43]; and base stacking energy (M12) [50]. For each data set, the rank of each classifier is shown in parentheses.

### Influence of negative data on performance estimates

To examine the influence of negative data on the estimated performance of predictors, we performed the following experiment. First, we decided to estimate the performance using blind test set experiments in order to avoid over-optimistic performance estimates reported using cross-validation experiments. Second, based on our suggested hypothesis, we picked up two classification methods: NB_DNID and NB_4-mer to represent good and bad predictors (respectively). Third, we trained these two classifiers using the seven versions of training data sets and tested them on the seven versions of blind test data. Tables 5 and 6 report the performance of NB_DNID and NB_4-mer in this experiment, respectively.

**Table 5:** AUC scores for Naive Bayes classifier with DNID features (NB_DNID) trained using seven versions of CV data and in each time tested on the seven versions of the independent test data.

Training data	TS_Random	TS_Coding	TS_Convergent	TS_Divergent	TS_CoPos	TS_CoNeg	TS_Mixed
CV_Random	0.87(2.0)	0.90(1.0)	0.80(5.0)	0.79(6.5)	0.79(6.5)	0.83(4.0)	0.84(3.0)
CV_Coding	0.80(2.5)	0.93(1.0)	0.74(6.5)	0.75(5.0)	0.74(6.5)	0.78(4.0)	0.80(2.5)
CV_Convergent	0.82(4.5)	0.84(1.0)	0.82(4.5)	0.80(6.5)	0.80(6.5)	0.83(2.5)	0.83(2.5)
CV_Divergent	0.80(4.5)	0.86(1.0)	0.80(4.5)	0.79(6.0)	0.77(7.0)	0.82(2.0)	0.81(3.0)
CV_CoPos	0.80(5.0)	0.84(1.0)	0.81(2.5)	0.80(5.0)	0.79(7.0)	0.80(5.0)	0.81(2.5)
CV_CoNeg	0.80(4.5)	0.86(1.0)	0.80(4.5)	0.79(6.0)	0.76(7.0)	0.83(2.0)	0.82(3.0)
CV_Mixed	0.83(2.5)	0.89(1.0)	0.80(5.5)	0.80(5.5)	0.79(7.0)	0.82(4.0)	0.83(2.5)
Average	0.82(3.6)	0.87(1.0)	0.80(4.7)	0.79(5.8)	0.78(6.8)	0.82(3.4)	0.82(2.7)
STD	0.03	0.03	0.03	0.02	0.02	0.02	0.01

Each row corresponds to a specified training set while each column corresponds to a specified test set.

**Table 6:** AUC scores for Naive Bayes classifier with 4-mer features (NB_4-mer) trained using seven versions of CV data and in each time tested on the seven versions of the independent test data.

Training data	TS_Random	TS_Coding	TS_Convergent	TS_Divergent	TS_CoPos	TS_CoNeg	TS_Mixed
CV_Random	0.87(1.0)	0.82(2.0)	0.63(5.0)	0.60(6.0)	0.58(7.0)	0.65(4.0)	0.69(3.0)
CV_Coding	0.73(2.0)	0.91(1.0)	0.59(6.0)	0.60(5.0)	0.56(7.0)	0.63(4.0)	0.68(3.0)
CV_Convergent	0.62(3.5)	0.56(6.5)	0.74(1.0)	0.56(6.5)	0.57(5.0)	0.64(2.0)	0.62(3.5)
CV_Divergent	0.64(4.5)	0.83(1.0)	0.61(6.0)	0.64(4.5)	0.55(7.0)	0.68(2.0)	0.66(3.0)
CV_CoPos	0.57(7.0)	0.65(2.0)	0.63(3.5)	0.59(6.0)	0.66(1.0)	0.61(5.0)	0.63(3.5)
CV_CoNeg	0.58(6.0)	0.74(1.0)	0.62(4.0)	0.60(5.0)	0.55(7.0)	0.71(2.0)	0.63(3.0)
CV_Mixed	0.76(2.0)	0.88(1.0)	0.64(5.0)	0.61(6.0)	0.58(7.0)	0.68(4.0)	0.70(3.0)
Average	0.68(3.7)	0.77(2.1)	0.64(4.4)	0.60(5.6)	0.58(5.9)	0.66(3.3)	0.66(3.1)
STD	0.11	0.13	0.05	0.02	0.04	0.03	0.03

Each row corresponds to a specified training set while each column corresponds to a specified test set.

Our first observation is that the coding regions should not be used to generate negative data for blind test sets. TS_Coding test set shows an over-optimistic performance of both classifiers. However, such low-quality test data can discriminate between good and bad predictors since NB_DNID seems to be less sensitive to the choice of training data (STD = ±0.03) while NB_4-mer seems to be more sensitive to the choice of training data version (STD = ±0.13).

For test sets with negative data generated from non-coding regions (e.g., TS_CoNeg, TS_CoPos, TS_Convergent, TS_Divergent), they are all successful in discriminating between good and bad predictors. Both NB_DNID and NB_4-mer have consistent good and poor performances (respectively) regardless which version of training data has been used for training the classifiers. TS_CoPos test set seems to be the most challenging test set because both classifiers have their lowest performance when this test data is used (see TS_CoPos columns in Tables 5 and 6).

For the test set with randomly generated sequences, Tables 5 and 6 show that TS_Random can always discriminate between good predictors (e.g., NB_DNID) and poor predictors (e.g., NB_4-mer) except when the two predictors are trained using CV_Coding or TS_Random. Therefore, results reported on blind test set where negative data are fragments of randomly generated DNA sequences should be handled with caution, especially when the negative training data was also generated using the same way.

Finally, for TS_Mixed where negative data has been obtained by mixing six subsets of equal numbers of negative data generated using the six approaches for generating negative data explored in this study, we noted that: i) TS_Mixed can successfully discriminate between good and bad predictors regardless which training data version is used for training the classifiers; ii) Performance of both classifiers is remarkably less sensitive to the type of training data (e.g., small STD value is reported for both classifiers).

In summary, any version of the training data sets (including the versions with randomly generated negative data and negative data extracted from coding regions) could produce a good classifier (e.g., a classifier with AUC score between 0.80 and 0.89 on the blind test set) when sequence data is represented using discriminative features (e.g., DNID features). On the other hand, test sets with negative data randomly generated or extracted from coding regions should be avoided.

### Comparison with existing prokaryotic promoter prediction servers

**Fig 3:**
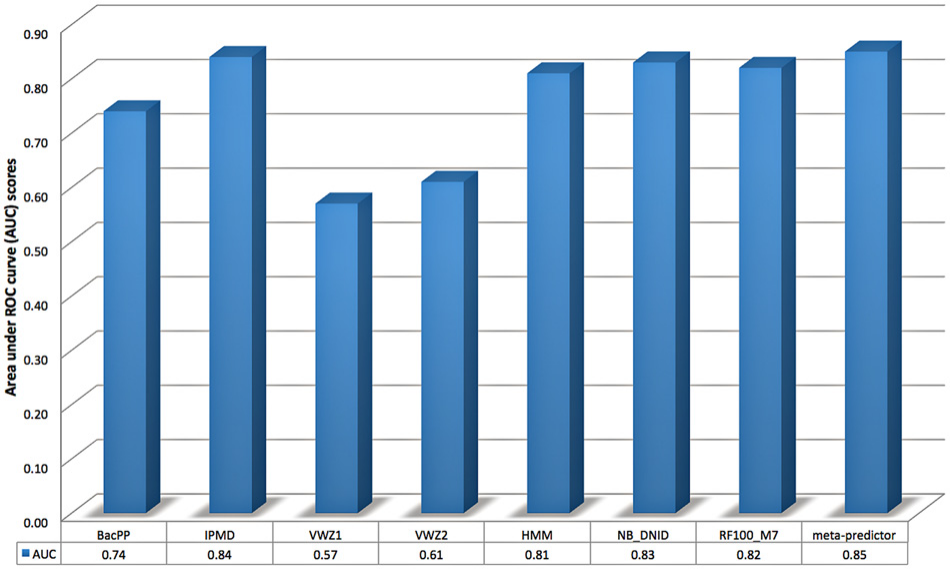
Performance comparison of BacPP, IPMD, and two variable-window Z-curve models, VWZ1 and VWZ2, trained using Datatset-1 and Datatset-2 (respectively) with four selected classifiers (NB_DNID, RF100_M7, HMM, and meta-predictor) using TS_Mixed independent test set.

Although our main goal is not to develop a predictor that outperforms the state-of-the-art methods for predicting prokaryotic promoters (in the sense that no attempts have been made to tune the parameters of any classifier considered in this study), it is of interest to figure out how the performance estimates of our identified good predictors compare with some existing methods for predicting *σ* ^70^ promoter regions in E. coli. To address this question, we compared NB_DNID_Mixed, HMM_Mixed, RF100_M7_Mixed, and meta-predictor classifiers trained using CV_Mixed data set with IPMD [15], BacPP [17], and variable-window Z-curve (VWZ) [18] methods. None of these methods has a Web server. However, the source code and the data sets used to evaluate VWZ method [18] can be downloaded at: http://www.csssk.net/publications. We adapted this code to return prediction scores instead of predicted binary labels and to train and test on two separate data sets instead of performing jackknife test on a provided data set. The modified code is provided in the Supporting Information section (S1 Code). For IPMD and BacPP methods, the TS_Mixed test set has been submitted to the authors of the two methods who kindly agreed to apply their methods to our test data and returned predicted probabilities to us. The predictions returned by these three methods were compared with the predictions of our four classifiers (in terms of AUC) in Fig. 3.

BacPP encodes nucleotides as 4-bit binary strings and uses artificial neural networks [66] for training its predictors using promoter data extracted from RegulonDB 6.1 and non-promoter sequences extracted from intergenic (non-coding) regions. The lacking performance of BacPP might be due to the low quality of the data set (e.g., insufficient training data and no attempts to remove highly similar sequence have been tried).

IPMD combines increment of diversity and position weight matrices for predicting eukaryotic and prokaryotic promoters. For training and evaluating the IPMD *σ* ^70^ promoter predictor, the authors used 1400 non-promoter sequences (700 coding and 700 convergent intergenic sequences). The observed IPMD performance (AUC = 0.84) is competitive with NB_DNID (AUC = 0.83) and meta-predictor (AUC = 0.85).

VWZ method extracts Z-curve descriptors [67] of k-mer features (for k = 1, …, 6) and uses a partial least squares (PLS) classifier combined with an iterative feature selection procedure to eliminate irrelevant and highly-correlated features. The method was evaluated on two data sets: Dataset-1 contains 576 *σ* ^70^ promoters (positive samples) and 836 coding fragments (negative samples) of E. coli; Dataset-2 contains the same 576 *σ* ^70^ promoters (positive samples) and 825 non-coding fragments (negative samples) of E. coli. Using jackknife tests, an excellent performance, accuracy ≥ 90% using top ranked 330 and 220 features for Dataset-1 and Dataset-2 (respectively) was reported [18]. Fig. 3 shows that the AUC scores for the two VWZ classifiers trained using Dataset-1 and Dataset-2 using optimal number of features and tested on TS_Mixed data are 0.57 and 0.61 (respectively). The huge discrepancy between the cross-validation (jackknife test is an *n*-fold cross-validation test, where *n* is the number of instances) performance reported in [18] and the independent test reported in this study might be in part justified by the low quality of the training data (i.e., no similarity reduction have been applied to Dataset-1 and Dataset-2). To test this hypothesis, we trained one more VWZ model using CV_Mixed data set and top ranked 220 features. The AUC for such model on the TS_Mixed test data is 0.70. This result emphasize the importance of independent test sets to confirm the performance estimates of cross-validation tests and to avoid misleading cross-validation results that might be observed due to the redundancy in the data or due to the possibility that some classifiers might overfit the data and produce a model with impressive cross-validation performance and a poor generalization performance on other independent data sets.

## Conclusions

The development of reliable prokaryotic promoter region prediction methods is highly desirable for improving the accuracy of microbial genomes annotation tools. A major limitation in developing reliable prokaryotic promoter region predictors is the lack of experimentally validated non-promoter data. We evaluated several strategies for generating non-promoter sequences and showed that a more accurate estimate of the classifier performance could be obtained using negative data consisting of equal size subsets of sequences generated using multiple strategies or by generating multiple versions of the cross-validation data (each with negative data generated with a different strategy) and use the average cross-validation performance over these data sets as the estimated cross-validation performance of the classifier. This approach would be very useful in cases where it is hard to obtain more experimental data to be used for independent test experiments. We also showed that a good predictor and/or good feature representation should allow for the discrimination between promoter sequences and all types of non-promoter sequences.

Cross-validation experiments are widely used for estimating the performance of classifiers developed for different bioinformatics classification tasks. In this work, we showed that for some combination of developers’ design decisions (e.g., randomly generated non-promoter sequences with k-mer features), cross-validation estimates might be misleading regarding the performance of the classifier. For example, a Naive Bayes classifier using 4-mer features has AUC = 0.87 on such cross-validation data, while its performance drops to AUC = 0.56 when evaluated using a high-quality test set. To avoid such misleading conclusions, independent test sets (when possible) should be used to evaluate the performance of the proposed prediction methods.

Sequence-based approaches for developing prokaryotic promoter region predictors are highly competitive to structure-based approaches evaluated in this study. However, a slight improvement in performance is observed when combining predictors based on the two approaches. More improvement could be obtained using: i) more sophisticated approaches for combining classifiers [68]; ii) building a single classifier using combined sequence and structure features and using feature selection algorithms to remove irrelevant or redundant features.

Finally, due to the lack of sufficient experimental data, we limited our experiments to E. coli *σ* ^70^promoter region predictions. Our future work aims at extending this work to cover other *σ* factors and explore the influence of the four factors considered in this study on the development of related DNA sequence prediction methods. Given that obtaining negative data is a challenge for most bioinformatics classification tasks, we conjecture that our findings apply not only to the problem of predicting E. coli *σ* ^70^ promoter regions but also to other bioinformatics sequence classification tasks.

## Supporting Information

S1 Dataset Data sets used in this study. (ZIP)

S1 Text Detailed results on cross-validation and independent test sets. (XLSX)

S1 Code Matlab code for evaluating variable-window Z-curve method. (ZIP)
